# Development of Moral Judgments in Impersonal and Personal Dilemmas in Autistic Spectrum Disorders from Childhood to Late Adolescence

**DOI:** 10.1007/s10803-022-05795-6

**Published:** 2022-11-27

**Authors:** Melanie Labusch, Manuel Perea, Rosa Sahuquillo-Leal, Isabel Bofill-Moscardó, Ángel Carrasco-Tornero, Antonio Cañada-Pérez, Ana García-Blanco

**Affiliations:** 1 Center for Research in Cognition, Nebrija University, Madrid, Spain; 2 Department of Methodology, University of Valencia, Valencia, Spain; 3 Neonatal Research Unit, Health Research Institute La Fe, Avda de Fernando Abril Martorell, 106, 46026 Valencia, Spain; 4 Department of Psychiatry and Clinical Psychology, University and Polytechnic Hospital La Fe, Valencia, Spain; 5 Biostatistics and Data Science Unit, Health Research Institute La Fe, Valencia, Spain; 6 Department of Personality, Evaluation and Psychological Treatment, Valencia, Spain

**Keywords:** Moral dilemma, Autistic spectrum disorders, Social cognition, Empathy, Dual-process theory

## Abstract

A potential underlying mechanism associated with the difficulties in social interactions in Autistic Spectrum Disorders (ASD) concerns the abnormal development of moral reasoning. The present study examined utilitarian and deontological judgments in impersonal and personal moral dilemmas, comparing 66 individuals with ASD and 61 typically developing (TD) individuals between 6 and 18 years. Utilitarian judgments decreased with age. This decline was much more gradual for personal dilemmas in the ASD than in the TD group. ASD individuals rated utilitarian judgments as more appropriate but felt less calm, consistent with the Empathy Imbalance hypothesis. Utilitarian judgments were associated with social interaction difficulties in ASD. These findings identify possible social therapeutic targets for more efficient coping strategies in individuals with ASD.

## Introduction

Individuals with Autistic Spectrum Disorders (ASD) are characterized by restricted interests, repetitive rigid behavior, and differences in communication and social interaction (American Psychiatric Association, 2013). Difficulties in interpersonal interactions and friendships are among the areas that most affect these individuals' daily functioning and quality of life (Deschamps et al., 2014). These difficulties may arise in part from differences in moral development and subsequent moral reasoning compared with individuals with typical development (TD) (e.g., see Bellesi et al., 2018; Gleichgerrcht et al., 2012; Schaller et al., 2019; Ros & Stokes, 2018). Indeed, it has been suggested that individuals with ASD exhibit more rational decision-making than TD individuals, including moral judgments in emotional scenarios (“enhanced rationality” hypothesis in ASD; see Rozenkrantz et al., 2021, for a recent review). The scarce literature on this issue has focused on adults with ASD; little is known about the development of moral reasoning from childhood to adolescence in ASD (see Dempsey et al., 2020, for a review). The present study aims to fill this gap by analyzing the influence of emotion on moral decision-making in individuals with ASD from childhood to late adolescence, thus providing valuable insights into their moral reasoning development.

The most studied dilemmas that examine the influence of emotion on moral decision-making are the trolley dilemma's impersonal and personal scenarios (Foot, 1967; Thomson, 1976). In the impersonal scenario (“switch dilemma”), the participant has to deal with a runaway trolley that cannot be stopped. The trolley would undoubtedly kill five people on the track unless a lever that diverts the trolley to another track is pulled. Critically, if diverted to the other track, only one person would be killed by the trolley. In the personal scenario (“footbridge dilemma”), the participant has to decide whether to push a large man off a footbridge to stop a train from killing five people on the track. In the personal and impersonal scenarios, the decision is the same: would you decide to sacrifice one person to save five others (i.e., utilitarian decision), or would you let the trolley hit the group of five people (i.e., deontological decision)? However, the two scenarios differ in emotional engagement: switching a lever conveys a low emotional charge (impersonal scenario), whereas pushing a person to her/his death implies a substantial emotional charge (personal scenario) (Greene et al., 2001). In the impersonal scenario, most children and adults with TD choose to pull the switch (i.e., the utilitarian decision). However, in the personal scenario, the majority of individuals with TD would not push the person onto the tracks, adopting a deontological decision instead (e.g., Greene et al., 2001; Moore et al., 2008; Pellizzoni et al., 2010). Indeed, most TD adults feel a strong emotional aversion to the utilitarian decision in personal dilemmas (e.g., Petrinovich et al., 1993; Skulmowski et al., 2014).

Rationalist theories posit that moral decision-making relies on cognitive reasoning to engage in both a cost–benefit analysis (Kohlberg, 1969) and an emotional reaction to others’ distress (Damasio, 1994; Turiel, 1983). Dual-process theories (Greene, 2009; Greene et al., 2001, 2004; Moll & de Oliveira-Souza, 2007) combined these concepts to understand moral judgments in impersonal and personal scenarios. On the one hand, controlled cognitive processes are usually involved in low emotionally charged moral dilemmas (i.e., impersonal scenarios). Therefore, they promote utilitarian judgments, which means deciding to approve a harmful action when it serves a greater good. On the other hand, intuitive, emotional processes are usually involved in high emotionally salient moral dilemmas (i.e., personal scenarios), promoting deontological judgments (i.e., the decision to favor a person's rights) even though they do not lead to a greater good.

Clearly, the development of moral maturity requires cognitive and emotional abilities. An important issue is whether this development is delayed or atypical in ASD. Indeed, differences in empathy (i.e., a response to another individual based on their psychological or contextual circumstances; Hoffman, 2001) among individuals with ASD may reflect delayed or atypical moral development (see Dempsey et al., 2020; Mathersul et al., 2013). Empathy can be distinguished into the cognitive empathy system (i.e., the ability to infer the internal mental state of another individual) and the affective empathy system (i.e., the capacity to automatically experience an appropriate emotional response to another individual's emotional state) (Blair, 2005, 2008). While individuals with ASD may have a lower cognitive empathy system than their TD peers, their affective empathy system appears to be preserved (Dziobek et al., 2008; Rueda et al., 2015) or even heightened (Baron-Cohen, 2009; Smith, 2009a)—this has been named the "empathy imbalance hypothesis" (Smith, 2009a). This is particularly problematic, as differences in empathy levels have been found to hurt the personal well-being of individuals with ASC (Ros & Stokes, 2018).

Regarding prior research on personal and impersonal dilemmas in ASD, three studies have examined the moral decisions in adults with ASD (e.g., Gleichgerrcht et al., 2012; Patil et al., 2016; Schaller et al., 2019). These studies showed that, in the impersonal switch dilemma, the percentage of utilitarian responses was similar for adults with ASD and adults with TD (e.g., 78% of adults with ASD and 72% of TD adults decided to pull the switch in the Gleichgerrcht et al., 2012, study; see also Patil et al., 2016, and Schaller et al., 2019, for converging evidence). The evidence of across-group differences in personal dilemmas in adults with ASD is not entirely conclusive. For instance, Gleichgerrcht et al. (2012) found that 36% of adults with ASD decided to push the person from the bridge, and this figure was reduced to 14% for TD adults. While Schaller et al. (2019) found, numerically, a similar pattern as Gleichgerrcht et al. (2012), Patil et al. (2016) found a non-significant difference in the opposite direction.

Importantly, Schaller et al. (2019) also examined the switch and footbridge dilemmas with a group of 16 adolescents with ASD and 22 with TD (14–18 years old). In the impersonal switch dilemma, adolescents with and without ASD showed a similar percentage of utilitarian responses (60% of adolescents with ASD, 57% of TD adolescents). In the personal footbridge dilemma, adolescents with ASD chose the utilitarian response more often (20%) than TD adolescents (9%). However, this difference did not reach significance, probably due to the small sample size. To explain their findings, Schaller et al. (2019) suggested that the differences in utilitarian decisions in moral dilemmas for individuals with ASD could reflect a delay in developing social skills that could extend to adulthood. Consistent with this view, Gleichgerrcht et al. (2012) reported an association between utilitarian decisions in moral dilemmas and difficulties in social skills for adult individuals with ASD.

Clearly, to fully understand the development of moral judgments in ASD, it is necessary to study a wide range of ages from childhood to late adolescence. Previous research on moral development in TD children has shown that children between 9 and 10 years old typically choose the utilitarian decision in moral dilemmas regardless of their emotional engagement (Bucciarelli, 2015). As children grow up, deontological judgments increase gradually because of an upturn in their cognitive and emotional resources and the capacity to represent and keep in mind the models of alternative options (see Bara et al., 2001; Bucciarelli, 2015; Bucciarelli et al., 2008). As adults and adolescents with ASD appear to show a different pattern of moral decisions in personal moral dilemmas than TD individuals (Gleichgerrcht et al., 2012; Schaller et al., 2019), the roots of this effect would presumably originate at a younger age.

Besides age, the underlying emotional and cognitive processes for the development of moral decision-making in ASD should be considered. To shed some light on these processes, previous research has assessed individuals’ ratings of the appropriateness of their decisions (i.e., a measure of cognitive reasoning) and how they felt about their decisions (i.e., a measure of emotional reaction). Gleichgerrcht et al. (2012) found that adults with ASD expressed that their utilitarian responses to the personal dilemma were inappropriate but felt less emotional arousal than TD participants. However, whereas Schaller et al. (2019) reported that the two groups did not differ in the personal dilemma, individuals with ASD rated their utilitarian decisions more permissible and felt less emotional arousal in the impersonal dilemma. Conversely, Patil et al. (2016) asked how emotionally arousing participants found the scenarios (not their decisions) and found that, regardless of the dilemma type, adults with ASD reported more emotional arousal than TD participants. These conflicting findings may be because individuals with ASD show constricted emotional functioning characterized by a difficulty in identifying and reporting their feeling states (i.e., alexithymia; see Griffin et al., 2016; Hill et al., 2004; Uljarevic & Hamilton, 2013). Prior research has shown that especially expressing their own emotions was particularly difficult for children with ASD relative to TD children (Costa et al., 2017; Lartseva et al., 2015). To minimize these interpretive issues, we chose pictograms to measure the individuals' emotional arousal and appropriateness in the present experiment. The reason is that pictograms have been considered more appropriate than verbal self-reports in ASD individuals (see Bird & Cook, 2013; Frith & Happé, 1999; Uljarevic & Hamilton, 2013) (see Appendix 1, for a depiction of the pictograms).

In sum, the present experiment applied two well-studied dilemmas (i.e., the impersonal switch dilemma and the personal footbridge moral dilemma) to children and adolescents with ASD. Thus, this research fills the gaps in knowledge about moral judgments because: (1) it includes young individuals with ASD ranging from childhood to late adolescence; (2) it includes assessing the individuals’ emotional arousal and the rating of acceptability of their moral decision employing pictograms; and (3) it includes a full assessment of ASD symptomatology (i.e., social interaction, language, communication, restrictive interests, and repetitive behavior), and their association with moral decisions. The predictions are as follows. First, taking as reference the model on moral development in TD children (e.g., Bucchiarelli, 2015) and the “enhanced rationality” hypothesis in ASD (Rozenkrantz et al., 2021), we expected a less steep decrease in utilitarian decisions as a function of age for ASD children than TD children, especially in the personal moral dilemma. Second, based on the empathy imbalance hypothesis in individuals with ASD (Smith, 2009a), we expected children and adolescents with ASD to feel more emotional arousal due to a heightened affective empathy (Baron-Cohen, 2009; Smith, 2009a) but, at the same time, to rate their utilitarian decision as more appropriate than TD children and adolescents due to a reduced cognitive empathy (Blair, 2008; Smith, 2009a). Third, based on the importance of social cognition for moral reasoning (see Bellesi et al., 2018; Gleichgerrcht et al., 2012; Schaller et al., 2019), we expect to find a positive correlation between social interaction difficulties and utilitarian judgments in personal moral dilemmas.

## Method

### Participants

A final sample of 127 children and adolescents between 6 and 18 years of age took part in the study. The participants were 66 ASD out-patients from the Department of Psychiatry and Clinical Psychology, and 61 children with TD recruited in two local primary schools comparable in sex, age, and Intelligence Quotient. All individuals in the clinical group fulfilled the DSM-5 criteria (American Psychiatric Association, 2013) for ASD. The demographic and clinical details are shown in Table 1.

**Table 1:** Demographic and clinical details for both the TD group and the ASD group

Variable	TD group	ASD group
	(n = 61)	(n = 66)
	Mean (SD) / n(%)	Mean (SD) / n(%)
	Median (1st, 3rd Q.)	Median (1st, 3rd Q.)
Age	11.39 (2.97)	11.94 (2.88)
	11 (9, 15)	11.5 (10, 14)
Sex		
Boy	53 (86.89%)	56 (84.85%)
Girl	8 (13.11%)	10 (15.15%)
K-BIT scores	101.23 (11.91)	104.79 (16.52)
Vocabulary subtest	102.34 (13.33)	106.45 (17.86)
Matrix subtest	103.21 (7.09)	104.41 (12.29)
ADI-R		
Social interactions	–	15.88 (5.14)
	–	15 (12, 21)
Language/communication	–	11.67 (4.8)
	–	11 (8, 15.25)
Repetitive behavior/interests	–	5.34 (2.76)
	–	5 (3, 7)
Early development	–	2.92 (1.66)
	–	3 (2, 4)
CBCL scores	17.43 (8.44)	64.7 (23.99)
Anxious/depressed	16 (10, 21)	66 (47, 78.5)
	1 (0, 2)	8.5 (6, 11)
Withdrawn/depressed	1.49 (2.28)	6.41 (3.43)
	1 (0, 1)	7 (3.5, 8)
Somatic complains	1.36 (1.33)	3.65 (2.65)
	1 (0, 2)	3 (2, 5)
Social problems	1.34 (1.33)	9.17 (3.67)
	1 (0, 2)	10 (6, 11.5)
Thought problems	0.79 (1.07)	6.65 (3.68)
	1 (0, 1)	7 (4, 9)
Attention problems	3.56 (2.16)	9.86 (4.23)
	3 (2, 4)	10 (7, 13.5)
Rule-breaking behavior	1.39 (1.38)	4.37 (3.39)
	1 (0, 2)	4 (2, 6)
Aggressive behavior	2.82 (2.38)	10.44 (6.53)
	2 (1, 4)	9 (4.5, 14)

Eligible participants from the ASD group were children and adolescents who had been diagnosed by the referring clinicians before the study. Additionally, a trained clinical psychologist confirmed ASD diagnosis by individually interviewing parents using the Autism Diagnostic Interview-Revised (Lord et al., 1994), which measuring scales are according to the DSM-5 criteria (Reciprocal Social Interactions, Language/Communication, Repetitive Behaviors/Interests*,* and evidence of onset before 36 months of age) (American Psychiatric Association, 2013).

Exclusion criteria were other psychiatric diagnoses based on the case note review in ASD children. TD children did not have a psychiatric history, as reported by their parents. Additionally, participants were excluded if they had a verbal IQ below 80 in the Kaufman Brief Intelligence Test (K-BIT; Kaufman, 1997), neurological history, major medical disorders, or medication use that could influence cognition (e.g., psychotropic medicines, treatment with corticosteroids).

**Fig. 1:**
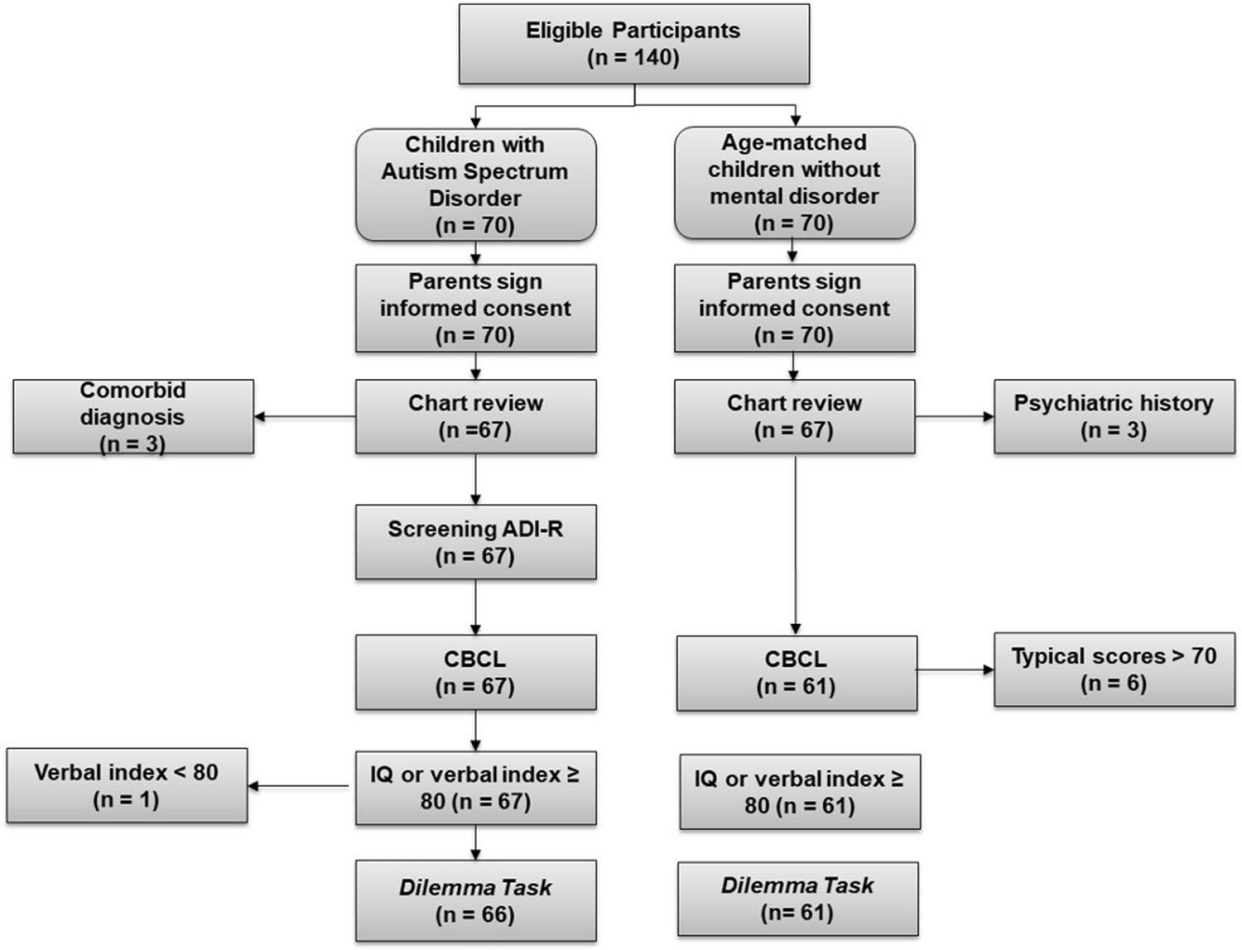
Flow diagram describing the recruitment process, the exclusion determinants, and the patients who completed the study

Furthermore, parents completed the Child Behavior Check List (CBCL; Achenbach, 1991) to control the subclinical symptomatology in TD children and the severity of any syndromes in ASD children. CBCL assesses information on problem behavior in children between 6 and 18 through eight syndrome scales (Anxious/Depressed, Withdrawal, Somatic Complaints, Social Problems, Thought Problems, Attention Problems, Rule-Breaking Behavior, Aggressive Behavior). A standardized T-score > 70 in the control group for any syndrome scale was considered an exclusion criterion. See Fig. 1 for the selection process.

### Procedure

After signing an informed consent form, parents answered to CBCL and ADI-R interview (in the case of ASD children). Children were assessed individually in a quiet room. Subsequently, the children received the non-moral dilemma (as a control condition) and the two moral dilemmas in a counterbalanced order. The text of the dilemmas is presented in Appendix 2. The moral dilemmas were the standard trolley dilemma and the footbridge dilemma (Greene et al., 2001, following Foot, 1967; Thomson, 1976). Participants had to choose whether to harm one person to save five people in both moral scenarios. After each decision, participants had to report on a 6-point Likert scale from 1 = totally inappropriate/completely restless to 6 = totally appropriate/completely calm: 1) if they thought their decision was appropriate [How appropriate is it to [take the train, switch the lever, push the person]?]”), and 2) if they were calm after their decision [“How calm do you feel about your decision?”]). The order of presentation of these questions was counterbalanced.

### Data Analysis

Continuous variables were summarized using the mean (standard deviation) and median (1^st^, 3^rd^ quartiles). Categorical variables were summarized using absolute and relative frequencies (in percentages). We created a Bayesian mixed logistic regression model on the utilitarian responses with the fixed factors Age, Group (ASD, TD), and Dilemma (personal, impersonal, neutral), including individuals as a random intercept. The model's reference levels were the neutral dilemma for the factor Dilemma and TD for the factor group. To assess the effect of group and dilemma on the two response variables after the decision (i.e., appropriateness, calmness), we created Bayesian mixed ordinal regression models with Appropriateness (or Calmness), Age, and Group as fixed factors, including individuals as a random intercept. A Bayesian logistic regression was conducted in the ASD group to assess the associations between utilitarian response and ADI-R. Weakly regularization priors were used for all fixed effects in the models. Interpretation of the results was performed using the 95% credible interval. An effect was considered significant when the 95% credible interval of its estimate (an Odds Ratio, OR) did not contain 1. All statistical analyses were performed using R (R Core Team, 2021) using the *brms* (Bürkner, 2017) and *clickR* (Fornes & Hervas, 2020) packages.

## Results

Descriptive data showing the percentage of utilitarian responses and their assessment in terms of appropriateness and calmness for each dilemma in each group are displayed in Table 2.

**Table 2:** Descriptive data from the assessment of appropriateness and calmness with the utilitarian decision for each dilemma in both groups

	Neutral dilemma		Impersonal dilemma		Personal dilemma	
	TD group	ASD group	TD group	ASD group	TD group	ASD group
Percentage of utilitarian responses	100%	95%	95%	100%	39%	92%
	Mean (SD)	Mean (SD)	Mean (SD)	Mean (SD)	Mean (SD)	Mean (SD)
	Median (1st, 3rd Q.)	Median (1st, 3rd Q.)	Median (1st, 3rd Q.)	Median (1st, 3rd Q.)	Median (1st, 3rd Q.)	Median (1st, 3rd Q.)
Appropriateness	5.15 (0.87)	5.13 (0.79)	3.67 (1.08)	3.93 (1.46)	3.25 (1.03)	3.77 (1.27)
	5 (5, 6)	5 (5, 6)	4 (3, 4)	4 (3, 5)	3 (3, 4)	4 (3, 4.5)
Calmness	5 (0.77)	5.15 (0.97)	3.16 (0.99)	3.09 (1.31)	3.42 (0.88)	2.88 (1.43)
	5 (4, 6)	5 (4.75, 6)	3 (3, 3)	3 (2, 4)	3 (3, 4)	3 (2, 4)

### Response to the Dilemmas

The estimates and the 95% credible intervals of the Bayesian mixed logistic regression model with the response (utilitarian vs. non-utilitarian [deontological]) as a dependent variable, and age, group, and dilemma are presented in Table 3—note that the 95% credible interval of the three-way interactions between dilemma, group and age did not contain 1. Since the two three-way interactions between dilemma, group, and age make the individual estimated parameters of the models difficult to interpret, we focused on partial dependence plots where these interactions can be easily understood. These plots also include 95% credible interval bands.

**Table 3:** Bayesian mixed logistic regression to assess the associations between utilitarian response and age, group, and dilemma—the significant effects are in bold

	Estimate	Std. Error	Exp (Estimate)	Lower 95%	Upper 95%
Age	1.12	0.98	3.06	0.7	29.90
ASD group	− 0.54	4.13	0.58	0	2267.36
Impersonal	4.04	4.30	56.64	0.01	275,047.64
Personal	− 1.33	3.67	0.26	0	415.76
Age: ASD group	− 1.33	0.97	0.27	0.03	1.14
Age: impersonal	− 1.71	0.95	0.18	0.02	0.71
Age: personal	− 2.44	1.17	0.09	0.01	0.498
ASD group: impersonal	0.35	4.94	1.42	0	21,235.28
ASD group: personal	2.91	3.78	18.43	0.01	27,941.72
Age: ASD group: impersonal	3.94	2.13	51.30	2.51	8783.73
Age: ASD group: personal	2.18	1.12	8.83	1.62	115.35

**Fig. 2:**
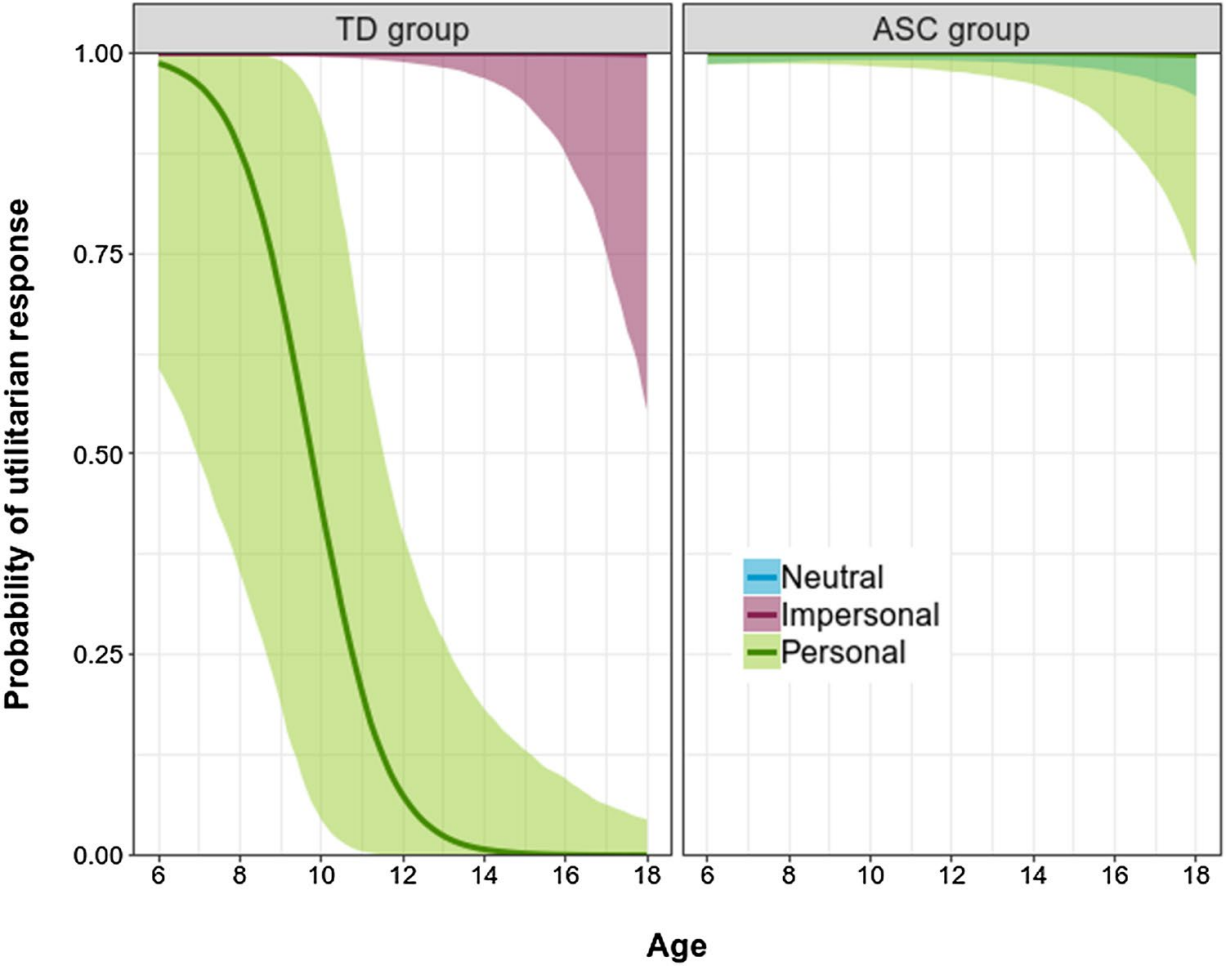
Partial dependence plots for the probability of a utilitarian response over age, separately for personal and impersonal dilemmas, for TD individuals (left panel) and individuals with ASD (right panel)

Figure 2 displays the partial dependence plot for the probability of utilitarian response over Dilemma, Age, and Group (left panel: TD group; right panel: ASD group). For younger children, the probability of a utilitarian response is high in both groups regardless of the dilemma. The key differences correspond to the interplay between age and group in the personal dilemma. As shown in Fig. 2, the effect of age on utilitarian responses in the personal dilemma is much less pronounced in the ASD group than in the TD group (OR = 8.83, CI 95% [1.62, 115]). Specifically, in the TD group, but not in the ASD group, the probability of utilitarian response in the personal dilemma sharply decreases as age increases (OR = 0.087, CI 95% [0.005, 0.49]).

### Appropriateness

**Fig. 3:**
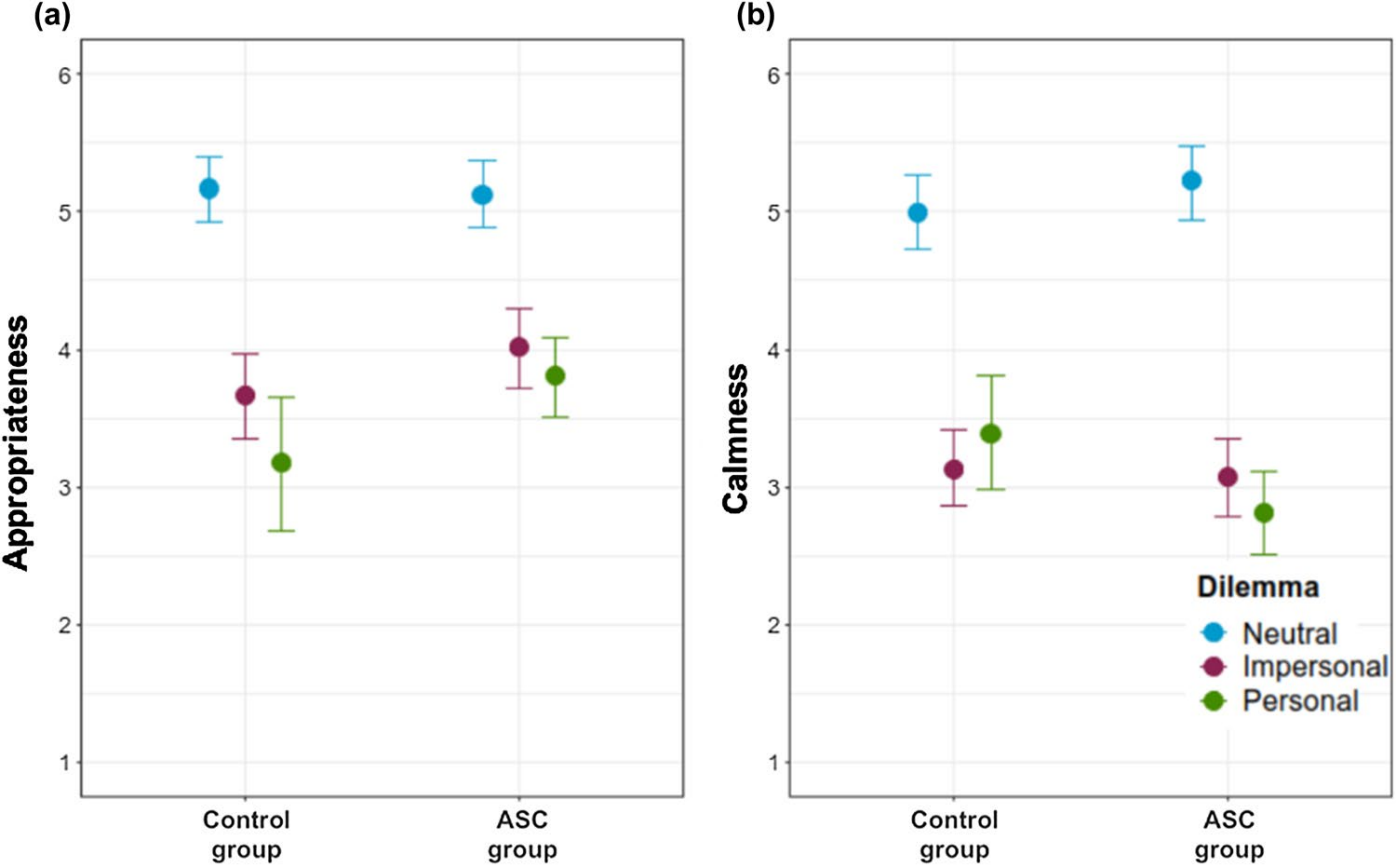
Appropriateness and calmness after utilitarian responses for each dilemma in each participant group

The estimates and 95% credible intervals for each of the effects of the Bayesian mixed ordinal regression to assess the effect of group and dilemma on appropriateness for the utilitarian responses are shown in Table 4. As can be seen in the left panel of Fig. 3, the ASD individuals who made a utilitarian response in the personal dilemma thought that their decision was more appropriate than the TD individuals (OR = 3.36, CrI 95% [1.12, 10.35]).

**Table 4:** Bayesian mixed ordinal regression to assess the effect of group and dilemma on appropriateness for utilitarian responses

	Estimate	Std. error	Exp(Estimate)	Lower 95%	Upper 95%
ASD group	− 0.112	0.402	0.894	0.412	1.971
Impersonal	− 3.035	0.398	0.048	0.022	0.104
Personal	− 3.884	0.53	0.021	0.007	0.057
ASD group: impersonal	0.748	0.488	2.113	0.824	5.449
ASD group:personal	1.214	0.575	3.368	1.117	10.354
sd(Intercept) code	1.112	0.21	–	0.708	1.524
WAIC	933.486	23.701

### Calmness

Table 5 displays the estimates and 95% credible intervals for each of the effects of the Bayesian mixed ordinal regression to evaluate the effect of group and dilemma on calmness for utilitarian responses. As shown in the right panel of Fig. 3, ASD individuals felt less calm than TD individuals (OR = 0.194, CrI 95% [0.062, 0.595]) after taking a utilitarian decision in personal dilemma.

**Table 5:** Bayesian mixed ordinal regression to assess the effect of group and dilemma on calmness for utilitarian responses

	Estimate	Std. error	Exp(Estimate)	Lower 95%	Upper 95%
ASD group	0.488	0.418	1.63	0.725	3.731
Impersonal	− 3.606	0.423	0.027	0.012	0.06
Personal	− 3.095	0.495	0.045	0.017	0.118
ASD group: impersonal	− 0.607	0.483	0.545	0.216	1.395
ASD group:personal	− 1.638	0.582	0.194	0.062	0.595
sd(Intercept) code	1.257	0.238	–	0.801	1.732
WAIC	925.63	24.886			

### ADI-R Scores and Moral Dilemmas

**Fig. 4:**
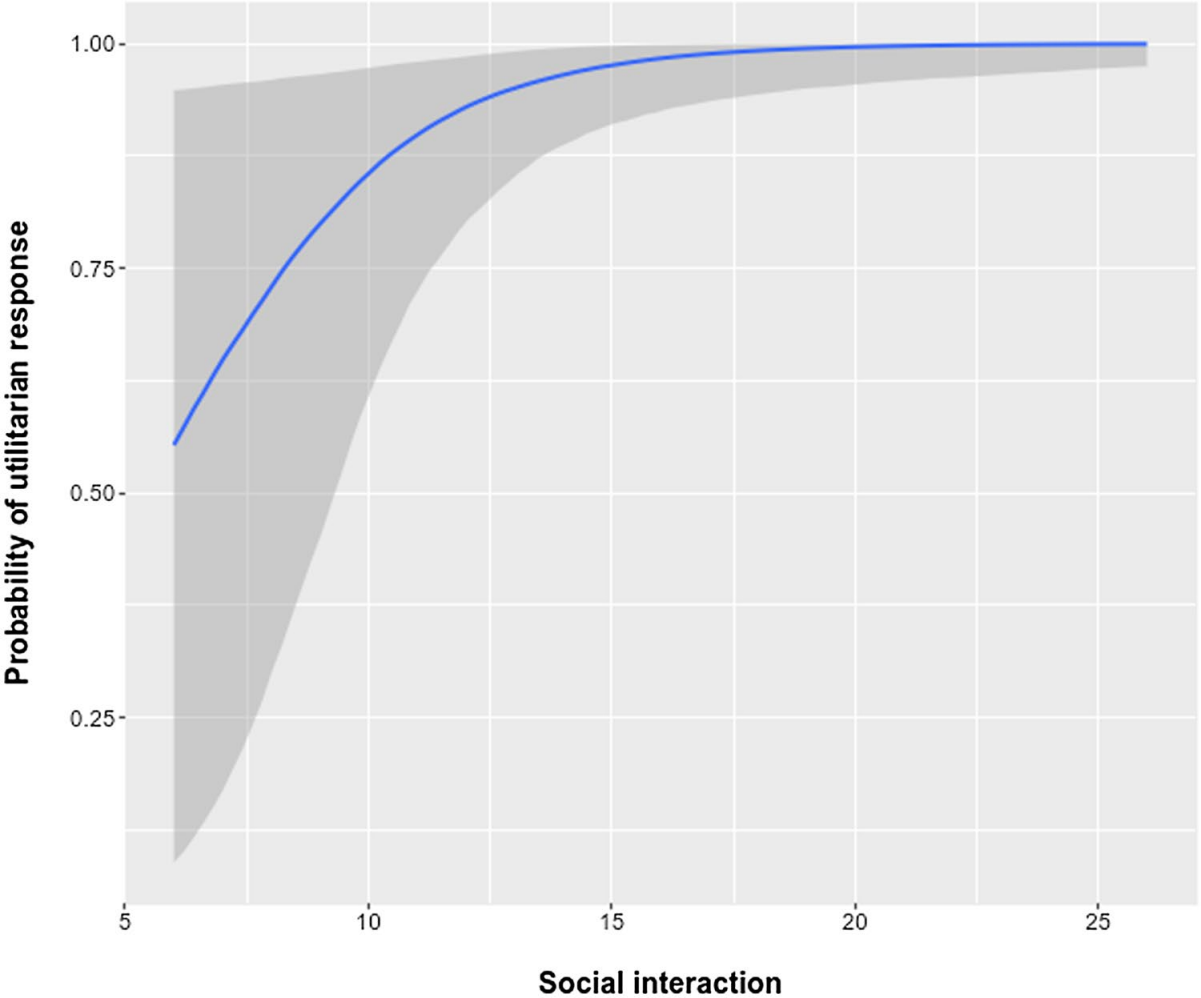
Association between the probability of a utilitarian response in the personal dilemma and the ADI-R social interaction score in the ASD group

Regarding the association between ADI-R scores in the ASD group and the probability of utilitarian responses for the personal dilemma, only the Social Interaction score showed a significant association (see Table 6). Specifically, the score on social interaction difficulties was related to the probability of utilitarian responses in the personal dilemma (OR = 1.49, CrI 95% [1.066, 2.266]; see Fig. 4, for depiction).

**Table 6:** Bayesian logistic regression for examining the associations between utilitarian response and ADI-R scores in the ASD group

	Estimate	Std. Error	exp(Estimate)	Lower 95%	Upper 95%
Intercept	− 1.83	1.955	0.16	0.003	6.278
Social interactions	0.398	0.19	1.49	1.066	2.266
Language/communication	0.033	0.163	1.033	0.75	1.443
Repetitive behavior/interests	− 0.034	0.268	0.967	0.577	1.64
Early development	− 0.179	0.337	0.836	0.43	1.618
WAIC	42.564	14.501

## Discussion

The present study examined the response to moral dilemmas (impersonal vs. personal) in a relatively large sample of children and adolescents with ASD vs. TD matched controls. As expected, we found that the probability of a utilitarian response decreased with age. Critically, this effect was modulated by group and type of dilemma (personal vs. impersonal). The probability of a utilitarian response decreased steeply with age for personal dilemmas for TD children. Conversely, the probability of a utilitarian response to the dilemmas barely decreased with age for children with ASD. Another notable finding is that, after making a utilitarian decision in a personal dilemma, the individuals with ASD not only rated their decisions as more appropriate than the TD controls but also felt less calm about their decisions. Finally, we found that choosing the utilitarian response in the personal dilemma in ASD individuals was associated with more difficulties in social interaction.

Regarding the moral development in ASD, we found more utilitarian decisions in the personal footbridge dilemma than in the impersonal switch dilemma, thus extending previous findings with adults (Gleichgerrcht et al., 2012; Patil et al., 2016) and adolescents (Schaller et al., 2019) to a children population. Critically, the inclusion of children and adolescents between the ages of 6 to 18 allows us to make inferences on moral reasoning development in individuals with ASD. First, the probability of a utilitarian response for 6-year-old children was nearly 100% for TD and ASD children. Second, in the range of 8 to 12 years of age, the probability of a utilitarian decision in a personal dilemma sharply decreased as age increased for TD children (see Bucchiarelli, 2015, for a model); however, this was not the case for ASD children. Indeed, for children with ASD, a decrease in the probability of a utilitarian decision in a personal dilemma only became visible from 14 years onwards. At the age of 18, although the probability of a utilitarian decision decreased with age in both groups, individuals with ASD showed a higher probability of a utilitarian response in a personal moral dilemma than TD individuals. Thus, the moral judgment of children with ASD develops atypically from 8 years of age onwards, in the way that it is delayed from the one of TD children. This delay relative to TD individuals remains at least up to young adulthood. These differences in moral judgments fit well with the “enhanced rationality” hypothesis (Rozenkrantz et al., 2021), which proposes that individuals with ASD use more rational and bias-free decision-making processes than TD individuals. Although one might argue that moral judgments could be hard to categorize in terms of rationality, utilitarian choices most often represent the option in which the greater good is maximized (i.e., most people are saved, despite the ethical consequences of the decision). Thus, an enhanced rational way of thinking in the development of ASD children may account for the increasing differences in moral decisions between young individuals with ASD and TD individuals in the present study.

Another novel feature of the present study was the individuals' evaluation of their choice (cognitive: appropriateness; emotional: calmness) after making a utilitarian judgment in the personal dilemma (i.e., pushing a person to death to save five others). While children with ASD rated their utilitarian decisions as more appropriate than the TD children, they also felt less calm about these judgments. The fact that individuals with ASD showed an incongruent assessment in these two domains favors those theories on ASD that posit that individuals with ASD have a decreased cognitive empathy system (Blair, 2008) but a heightened affective empathy system (Baron-Cohen, 2009; Smith, 2009a). Of note, previous studies showed conflicting findings on the rating of appropriateness and calmness in individuals with ASD, possibly due to difficulties in expressing the felt emotions (Costa et al., 2017; Lartseva et al., 2015). To assist the children with ASD, we chose pictograms to measure the individual level of calmness and appropriateness of the answers, so that possible difficulties in the expression of emotions merely have a minimal effect on our data.

Further evidence for a heightened emotional reaction to others’distress among individuals with ASD is coming from neuroimaging studies. During affective empathy tasks, a hyper-functioning of the amygdala occurs in individuals with ASD (Baron-Cohen et al., 2000), which, in turn, prompts individuals with ASD to act in a hyper-reactive way in social and emotional contexts (Intense World Hypothesis; Markram, Rinaldi, & Markram, 2007). This overstimulation would be reflected as less calmness after making a utilitarian response in ASD individuals than in TD controls. Consequently, when confronted with an emotionally charged moral decision (especially the case in personal moral dilemmas), a hyper-functioning affective empathy system would lead to overwhelmingly intense processing of the situation (Baron-Cohen et al., 2000). Following the Intense World Theory, a defective bottom-up modulation also has paradoxical cognitive consequences (Markram & Markram, 2010). Due to the excessive distress in potentially overwhelming situations, individuals with ASD may tend to withdraw from them by seeking less emotionally charged reasoning (Markram et al., 2007; Smith, 2009a, 2009b). In this way, individuals with ASD may manage to avoid overwhelming emotional processes by choosing a utilitarian decision that is interpreted as more “appropriate” (i.e., it saves the most lives). The current findings lead to new insights into empathy processing in individuals with ASD. Following the idea of the “double empathy problem” (Milton, 2012), the current findings help to reshape the view that a different empathy processing in ASD individuals is seen as a “deficit”, but rather as a different way of perceiving empathy and social interactions (Milton, 2012).

The present study also revealed that more reported social interaction difficulties in children/adolescents with ASD were associated with a higher probability of a utilitarian response in the personal moral dilemma. Thus, the high choice of utilitarian judgments in emotional scenarios may be related to the abnormal behavior in situations such as emotional sharing, offering and seeking comfort, social smiling, and responding to other children, which is often observed in individuals with ASD (Lord et al., 1994). In sum, the correlation between social interaction difficulties and utilitarian responses in children and adolescents with ASD extends previous evidence with adults. For instance, Gleichgerrcht et al. (2012) reported that, in personal moral dilemmas, a higher probability of utilitarian decisions was associated with more difficulties in social cognition in adults with ASD.

To our knowledge, the present study is the first that examined the development of moral judgments in individuals with ASD ranging from childhood to late adolescence, thus filling the gaps in knowledge about young individuals with ASD. Another novel element is that we measured the participants' emotional and cognitive states after making a utilitarian decision. Despite the strengths of our study, certain limitations have to be taken into consideration. First, we investigated moral judgments in children and adolescents using a cross-sectional rather than a longitudinal design. We chose this option because a longitudinal study from early childhood to late adolescence would have interpretive issues (e.g., same dilemmas at multiple times) and potential methodological problems (e.g., participants' dropping out of the study). Second, our study only employed two moral dilemmas: the personal “footbridge” dilemma and the impersonal “switch” dilemma. Although these are by far the two most studied moral dilemmas and serve as the basis for leading theories of moral judgments (e.g., Greene et al., 2001, 2004), including a wider variety of moral dilemmas in future research would have led to greater generalizability (e.g., see Patil et al., 2016).

Altogether, the present study revealed that emotion-based decision-making in ASD individuals does not develop similarly to TD individuals (i.e., probability of a utilitarian response in a moral dilemma decreased much more shallowly with age for children with ASD). Moreover, ASD individuals also show differences in the underlying cognitive beliefs and emotional responses to their behavioral decision. This difference was particularly manifest in an emotionally charged scenario (i.e., pushing a person from the bridge to save five lives). Notably, individuals with ASD felt less calm than their TD peers (exaggerated affective empathy system) after choosing a utilitarian decision, but they rated their utilitarian decisions as more appropriate (less developed cognitive empathy system). This finding suggests that by deciding on a utilitarian course of action 'for the greater good', those with ASD would place themselves in a position that caused them greater distress due to their awareness of the affective consequences of their decision. Furthermore, utilitarian judgments in personal dilemmas were related to more social interaction difficulties. Thus, therapeutic interventions that aim to improve the social skills of children with ASD should focus on both the development of their cognitive empathy system and the design of efficient coping strategies for emotionally charged situations.

## Figures and Tables

**Fig. 5:**
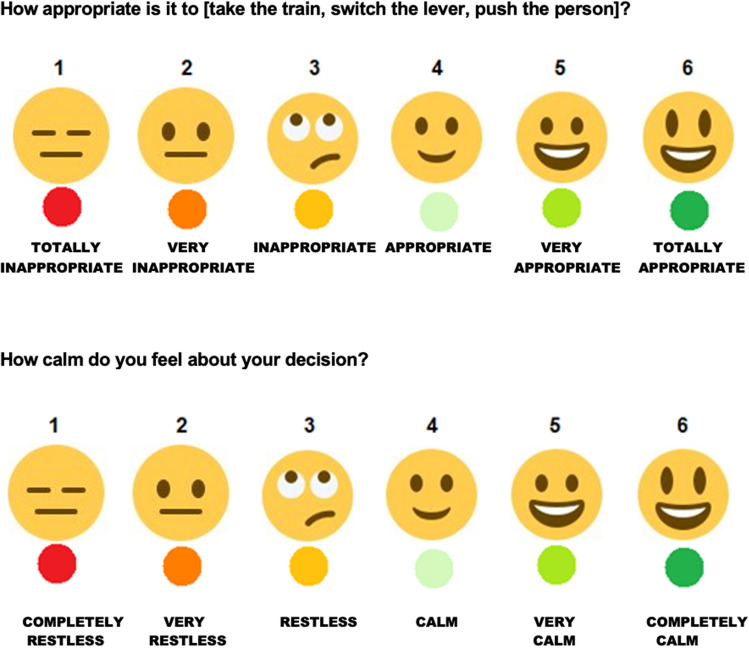
Examples of the pictograms that were used for the self-report

## Appendix 1. Example of pictogram for self-report

See Fig. 5

## Appendix 2. Text of the dilemmas used in the study

- *Trolley dilemma* A runaway trolley is coming down a railway track. The trolley is approaching five persons in a sidetrack who will not be able to leave in time before the trolley will hit them. If the trolley will continue it will certainly overrun the five persons and will kill all of them. You are standing beside a switch of a railway track. The only way for you to save the five persons is to throw the switch. This will cause the trolley to go to another track where it will overrun and kill one person, while the five persons on the first track will survive. Would you decide whether to flip a switch to redirect a trolley onto the man or whether to allow the trolley to hit the five people?
- *Footbridge dilemma* In this scenario, the trolley is approaching five persons on the railway track. This time you are on a bridge above the track, and a man is beside you. The only way for you to save the five persons is to push the man off the bridge so that his body would stop the trolley from hitting five people further down the tracks. Would you decide whether to push the man off the bridge or whether to allow the trolley to hit the five people?
- *Neutral dilemma* You are traveling from Valencia to Barcelona in order to attend a very important meeting that starts at 12:00. To travel, you can take either the train or the bus. The train will get you there just in time for the meeting: 5 min before. The bus is scheduled to arrive one hour before the meeting, but can sometimes be several hours late because of traffic. It would be great to arrive an hour before the meeting and walk around town, but you can't afford to be late. Would you take the train instead of the bus in order to guarantee you won't be late for the meeting, even if you arrive just in time?
